# 3D-Bioprinted Gelatin Hydrogels with Human Umbilical Cord Mesenchymal Stem Cell-Derived Small Extracellular Vesicles Promote Cutaneous Wound Healing In Vivo

**DOI:** 10.3390/polym18070882

**Published:** 2026-04-03

**Authors:** Manal Hussein Taghdi, Ibrahim N. Amirrah, Nurul Izzati Uda Zahli, Kavita Chirara, Mh Busra Fauzi, Jia Xian Law, Yogeswaran Lokanathan

**Affiliations:** 1 Department of Tissue Engineering and Regenerative Medicine, Faculty of Medicine, Universiti Kebangsaan Malaysia, Cheras, Kuala Lumpur 56000, Malaysia; manalhussein240@gmail.com (M.H.T.); noramirrahibrahim@gmail.com (I.N.A.); fauzibusra@ukm.edu.my (M.B.F.); lawjx@ukm.edu.my (J.X.L.); 2 Department of Anaesthesia and Intensive Care, Faculty of Medical Technology, University of Tripoli, Tripoli P.O. Box 13932, Libya; 3 Department of Veterinary Pathology & Microbiology, Faculty of Veterinary Medicine, Universiti Putra Malaysia (UPM), Serdang 43400, Selangor, Malaysia; nurulizzati.udazahli@upm.edu.my; 4 Department of Biotechnology, Faculty of Applied Sciences, UCSI University, No. 1, Jalan Menara Gading, UCSI Heights (Taman Connaught), Cheras, Kuala Lumpur 56000, Malaysia; kavita@ucsiuniversity.edu.my; 5 Advance Bioactive Materials-Cells UKM Research Group, Universiti Kebangsaan Malaysia, Bangi 43600, Selangor, Malaysia

**Keywords:** exosome, genipin, regeneration, angiogenesis, extracellular matrix, hydrogels

## Abstract

Small extracellular vesicles (sEVs) derived from mesenchymal stem cells (MSCs) are emerging as potent acellular therapeutics; however, their rapid clearance hinders their clinical translation. To address this issue, 3D-bioprinted genipin-crosslinked gelatin (GECL) was engineered for human health. GECL hydrogels were functionalised with human umbilical cord MSC-derived sEVs (hUCMSC-sEVs) to create a bioactive wound-healing platform. These hydrogels demonstrated favourable physicochemical, mechanical, and biodegradable properties while providing an extracellular matrix (ECM)-mimetic environment conducive to tissue regeneration. MSCs were isolated from the umbilical cords, and their small extracellular vesicles (sEVs) were extracted and incorporated into gelatin-based hydrogels via 3D bioprinting. These sEV-loaded scaffolds were embedded in full-thickness wounds in mice, and healing was evaluated through macroscopic observation, histological analysis, collagen deposition, and angiogenesis assessment. Compared with the untreated controls, both the hydrogel-only (B) and sEV-loaded hydrogel (BE) groups significantly accelerated in vivo wound healing. Notably, the BE group achieved complete wound closure within 14 days, restoring the skin architecture, which closely resembled the native tissue with well-organised epidermal and dermal layers, optimal thickness, and skin appendages. Histological and ultrastructural assessments revealed an increased collagen type I deposition, a reduced α-smooth muscle actin (α-SMA) expression, and a robust neovascularisation. The TEM revealed tight junctions and active cellular infiltration, indicating scaffold integration and functional remodelling. Immunohistochemistry further revealed an upregulated CD31 expression with a balanced α-smooth muscle actin (α-SMA) expression, reflecting coordinated angiogenesis and myofibroblast regulation. These results highlight sEV-functionalised GECL hydrogels as robust and clinically translatable acellular therapeutic green products for accelerated wound closure and functional skin regeneration, advancing the fields of regenerative medicine and life expectancy.

## 1. Introduction

Full-thickness wounds, involving the complete loss of the epidermis and dermis, remain a significant clinical challenge, particularly in patients with diabetes or infections due to impaired healing and who are at a high risk of scarring [1]. While conventional treatments often fall short, biomaterial-based therapies that incorporate stem cell derivatives offer promising alternatives. hUCMSCs are an attractive stem cell source because of their accessibility, low immunogenicity, and regenerative potential [2]. Secreted sEVs contain bioactive cargos (mRNAs, miRNAs, and proteins) that mimic the paracrine effects of MSCs without the risks associated with cell-based therapy [3]. However, the therapeutic effect of sEVs is limited by their rapid clearance in vivo [4], necessitating the use of a delivery vehicle that prolongs their presence and bioactivity.

Hydrogels are ideal for this application. They provide a moist 3D microenvironment conducive to cell migration and tissue repair [5]. When crosslinked with genipin, gelatin-based hydrogels offer tunable mechanical properties and stability [6]. The crosslinking mechanism involves a nucleophilic attack of gelatin’s primary amino groups on the olefinic carbon of genipin, initiating a ring-opening reaction that forms stable covalent networks. Under oxygenated conditions, secondary reactions generate water-soluble blue pigments, accounting for the characteristic bluish-green colouration of genipin-crosslinked hydrogels [7]. Moreover, 3D bioprinting allows for precise spatial control, enabling the fabrication of scaffolds that are tailored to the wound geometry [8,9]. The recent advances in 3D bioprinting have established it as a powerful and versatile technology for fabricating hydrogel-based scaffolds in tissue engineering and wound healing. Compared to conventional methods, bioprinting enables a precise spatial control of bioink deposition, allowing the creation of complex, biomimetic structures with customizable architectures and compositions. This capability facilitates the incorporation of cells and bioactive molecules, such as extracellular vesicles (EVs), to enhance the regenerative outcomes. In recent years, various biopolymer-based bioinks—including hyaluronic acid derivatives, gelatin methacryloyl (GelMA), alginate, and silk–alginate composites—have been explored for wound healing applications. These systems have demonstrated promising results, such as improved angiogenesis, enhanced tissue regeneration, and the potential for controlled EV delivery. However, several limitations remain, including insufficient mechanical strength, rapid degradation, and challenges in maintaining sustained bioactivity and release profiles. Despite these challenges, the integration of EVs into bioprinted hydrogel scaffolds represents a promising strategy to improve therapeutic efficacy. These advances highlight the need for optimised scaffold designs, such as the GECL system investigated in this study, to overcome current limitations and further enhance the wound healing outcomes [10].

In this context, tissue-engineered skin substitutes (TESS) have emerged as a promising strategy for wound management by providing structural support and biological cues that facilitate tissue repair. The acellular hydrogel-based scaffolds are particularly attractive due to their high-water content, biocompatibility, and ability to mimic the native ECM, thereby promoting cell adhesion, proliferation, and migration. The recent studies have highlighted the potential of bioprinted hydrogel scaffolds as bioactive platforms capable of enhancing wound healing and tissue regeneration [11].

Our previous study reported the development of 3D bioprinted genipin-crosslinked gelatin (GECL) hydrogels with physicochemical properties that are suitable for cutaneous wound healing [12]. The 10% GECL formulation exhibited optimal printability, mechanical strength, and degradation behaviour, rendering it suitable as a scaffold for skin tissue engineering. In this study, we integrated hUCMSC-sEVs into a 3D bioprinted 10% GECL scaffold and evaluated its regenerative efficacy in vivo. This study hypothesised that the sEV-loaded hydrogel would accelerate wound closure, enhance angiogenesis, and promote dermal remodelling more effectively than the hydrogel alone or the untreated controls in vivo. While our previous study focused on physicochemical optimisation and in vitro validation, the present work advances this platform by providing the in vivo evidence of its therapeutic efficacy and elucidating its effects on angiogenesis, extracellular matrix remodelling, and myofibroblast regulation.

## 2. Materials and Methodology

### 2.1. Ethical Approval

**Figure 1:**
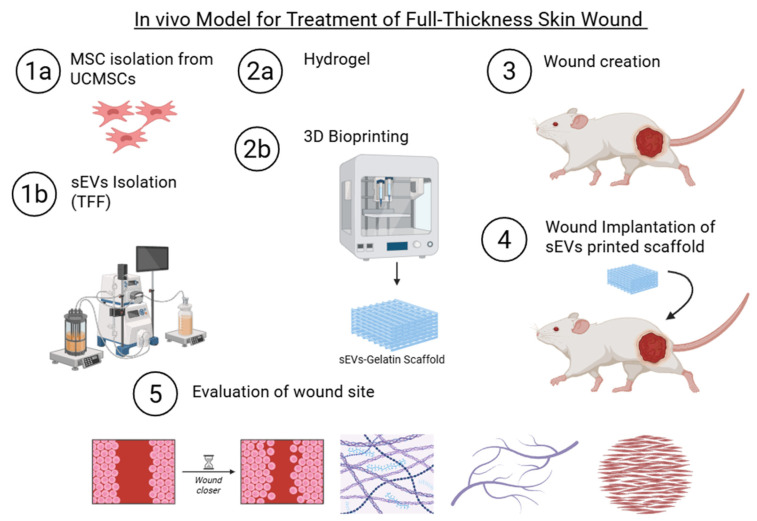
A structured five-step workflow was used in this study to develop and evaluate human umbilical cord mesenchymal stem cell-derived small extracellular vesicles (hUCMSC-sEVs) incorporated into three-dimensional (3D) bioprinted gelatin hydrogels for in vivo wound healing assessment. In Step 1, MSCs were isolated from the umbilical cord (1a), followed by the extraction of fresh sEVs via tangential flow filtration (TFF) (1b). Step 2 involved the preparation of gelatin-based hydrogels (2a) and the subsequent 3D bioprinting of sEV-loaded gelatin scaffolds (2b). In Step 3, full-thickness wounds were surgically created on the dorsal upper limbs of the mice. Step 4 involved the implantation of bioprinted scaffolds into the wound sites. Finally, Step 5 involved the evaluation of wound healing outcomes through macroscopic observation, histological analysis, collagen deposition, and angiogenesis assessment. The images were created using BioRender.com.

Figure 1 presents a schematic overview of the experimental design used in this study. The study received an animal ethics approval from the Animal Ethics Committee of Universiti Kebangsaan Malaysia (UKM) under the Research Ethics Committee, with the approval code JEP-2024-192.

### 2.2. Isolation and Characterisation of hUCMSC-sEVs

hUCMSCs at passage 5 were cultured in a serum-free medium for sEV collection and harvested at 70–80% confluency, with routine mycoplasma testing confirming the cell quality. For the in vivo experiments, sEVs were freshly isolated from the conditioned media using sequential centrifugation followed by tangential flow filtration (TFF) with a 100 kDa membrane, in accordance with the MISEV2023 guidelines. To summarise key characterisation parameters, particle size and concentration were measured by nanoparticle tracking analysis (NTA), confirming a size range of 50–200 nm, and vesicle morphology was verified by transmission electron microscopy (TEM). The total protein content was quantified using a BCA assay, and Western blotting confirmed the presence of canonical sEV markers (CD63, TSG101) and the absence of the negative marker GP96, ensuring the sample purity. All the procedures maintained sterility and hydration, and the preparation was consistent with our previously published characterisation, confirming the quality and reproducibility of the sEV batches [12]. As reported in our previous study, the 10% genipin-crosslinked gelatin (GECL) hydrogel encapsulating 75 µg/mL hUCMSC-sEVs demonstrated multifunctional properties and excellent biocompatibility, supporting its suitability for wound-healing applications [12].

### 2.3. Animal Management

Twelve male BALB/c mice aged eight weeks and weighing approximately 18–30 g were obtained from the Laboratory Animal Resource Unit, Universiti Kebangsaan Malaysia (UKM), Bangi, Malaysia. The animals were housed individually in individually ventilated polycarbonate cages (Allentown, Inc., Allentown, NJ, USA) under specific pathogen-free (SPF) conditions. The environmental parameters were maintained at a constant temperature of 22 ± 2 °C with a 12 h light/dark cycle. The mice were acclimatised for one week before the initiation of the experiment. Throughout the study, the animals had ad libitum access to autoclaved tap water and a standard rodent chow diet (Altromin 1314, Lage, Germany). The bedding, water, and feed were replaced daily. The housing environment ensures proper humidity, ventilation, and circadian rhythm regulation to support animal welfare and minimise stress.

### 2.4. Animal Grouping and Experimental Design

Nine healthy adult male BALB/c mice were used in this study. Each mouse was housed individually in an individually ventilated cage (IVC, Greenville, SC, USA; Biobubble, Fort Collins, CO, USA) under standard laboratory conditions with free access to food and water. The animals were randomly assigned to three experimental groups, resulting in six wounds per group (*n* = 6 wounds). Each mouse received two full-thickness excisional wounds that were created at anatomically separated dorsal sites, yielding a total of 18 wounds. The use of two wounds per animal was intended to reduce inter-animal biological variation while maintaining consistent experimental conditions. The experimental groups were as follows:

C group (control): the wounds were treated with 10 μL sterile normal saline and covered with sterile gauze.

B group (bioprinted scaffold): the wounds were treated with a 3D-bioprinted hydrogel scaffold composed of 10% (*w*/*v*) gelatin crosslinked with 0.3% (*w*/*v*) genipin (GECL) without sEVs and covered with sterile gauze.

BE group (sEV-loaded scaffold): the wounds were treated with a 3D-bioprinted gelatin–genipin scaffold incorporated with 75 µg/mL hUCMSC-derived small extracellular vesicles (sEVs) and covered with sterile gauze.

### 2.5. 3D Bioprinting and Scaffold Preparation

The sterile hydrogel scaffolds were fabricated using an extrusion-based 3D bioprinter (Biogen XI, 3D Gens, Shah Alam, Malaysia) equipped with a pneumatic print head and a temperature-controlled platform. A sterile 10% (*w*/*v*) gelatin solution was crosslinked with 0.3% (*w*/*v*) genipin at a ratio of 3:1 to produce the GECL scaffold. These concentrations were selected based on our previous work [12], which systematically evaluated hydrogel printability, mechanical stability, and biocompatibility. They provide an optimal balance between the scaffold structural integrity, the extrusion performance during the 3D bioprinting, and the suitability for cell and tissue compatibility in wound healing applications. For the BE group, small extracellular vesicles (sEVs) were incorporated into the hydrogel at a concentration of 75 μg/mL prior to printing.

Each hydrogel formulation was loaded into a sterile 10 mL syringe fitted with a 400 μm nozzle. The circular scaffold models were designed using Autodesk Fusion 360 and were exported as STL files. The printing paths were generated using Simplify3D software (v4.1, Simplify3D, Cincinnati, OH, USA). The printing was performed at a maintained nozzle temperature of 25 °C to ensure an optimal material extrusion. The cylindrical scaffolds with a diameter of 1.0 cm and a thickness of 0.5 mm were fabricated using a layer-by-layer printing strategy to form four-layered structures.

All the hydrogel scaffolds were fabricated under sterile conditions immediately prior to implantation, maintaining their sterility and hydration throughout handling. No post-printing storage or additional sterilisation was performed, and the same well-established preparation procedures were applied consistently as in our previous in vitro work [12].

### 2.6. Anaesthesia and Wound Creation

The anaesthesia was induced using a well-established anaesthetic cocktail (KTX), which was prepared based on the method described by [13]. The KTX mixture included 250 mg Zoletil 50 (Virbac, Carros, France), 2.5 mL ketamine 100 (250 m, Mavlab, Slacks Creek, Australia), and 12.5 mL Xylazil-20 (250 m, Troy Laboratories, Glendenning, Australia). The final concentrations were 16.67 mg/mL of ketamine and xylazine and 8.33 mg/mL of tiletamine and zolazepam. The mice received 0.1 mL of this solution per 10 g of body weight via intramuscular injection. Once anaesthetised, the upper hind limb region was shaved, disinfected with 70% ethanol (Sigma-Aldrich, St. Louis, MI, USA), and marked with a 10 mm biopsy punch (Kai Medical, Seki, Japan) to define a circular wound (~0.79 cm^2^). A full-thickness excisional wound was created using surgical scissors.

### 2.7. Wound Treatment and Monitoring

The printed scaffolds were aseptically implanted into the wound beds and secured using five evenly spaced absorbable 3/0 sutures (Otosilk^TM^, Covington, GA, USA). The wounds were dressed with sterile gauze and fixed with Hypafix adhesive tape (BSN Medical, Hamburg, Germany). All the dressings were changed on day 7. The wound healing progression was monitored and photographed using a digital camera on days 0, 7, 10, and 14. The gross wound appearance was used to evaluate the healing efficiency. At the end of the study, fully closed wounds were harvested for further analysis.

### 2.8. Safety Monitoring

Animal welfare was monitored throughout the study. The body weight, behaviour, and vital signs were recorded regularly. Pain, discomfort, inflammation, and signs of allergic reactions (e.g., rash, swelling, and hypersensitivity) were also observed. The wound sites were examined for complications such as infection, delayed healing, or scarring, ensuring the safety and biocompatibility of the implanted scaffolds.

### 2.9. Gross Appearance Evaluation

The area of the wound was grossly captured using a digital camera on days 0, 7, 10, and 14 using a measurement scale. The area of the wound at each time point was analysed using ImageJ software (v1.53t; NIH, Bethesda, ML, USA), and the percentage of the wound area was calculated as follows:

Wound closure (%) = (W_0_ − W_t_/W_0_)100

where W_0_ is the initial wound area, and W_t_ is the wound area at each time point.

### 2.10. In Vivo Biodegradation Assessment of the Scaffolds

In vivo scaffold biodegradation and biocompatibility were evaluated in adult male BALB/c mice (8 weeks old, *n* = 3 per group). The mice were anaesthetised intramuscularly with ketamine (50 mg/kg, Mavlab, Australia), xylazine (5.6 mg/kg, Troy Laboratories, Australia), and Zoletil^®^ (20 mg/kg, Virbac, France). A 1 cm dorsal incision was made, and subcutaneous pockets were created for sterile hydrogel discs (diameter 1 cm, thickness 0.5 cm), which were implanted, and the wounds were closed with absorbable sutures.

The mice were assigned to the B hydrogel *(n* = 3) and BE (*n* = 3) groups. The scaffold dimensions (length, width, and height) were measured with a digital calliper on days 0, 5, 7, 10, 12, and 14 to monitor volume changes. The volume was calculated as:

Volume = Length × Width × Thickness

The biodegradation rate was calculated via the following formula:

Biodegradation Rate = V_0_ − V_t_/t

where V_0_ is the initial volume (mm^3^), V_t_ is the volume at time t (mm^3^), and t is the time elapsed (days).

On day 14, the animals were sacrificed, and the surrounding tissues were collected and fixed in 4% paraformaldehyde for histological evaluation via H&E staining. The scaffold biodegradation was assessed both macroscopically and histologically at predefined sites, with the residual scaffold evaluated qualitatively in a blinded manner.

### 2.11. Sample Collection and Histological Evaluation

Upon completion of the study (day 14), the mice were euthanised via an overdose of anaesthetic, followed by cervical dislocation. A skin sample of approximately 1 cm in diameter, including the wound area, was excised. The samples were fixed in 10% formalin for histological and immunohistochemical (IHC) evaluation and 4% glutaraldehyde for ultrastructural analysis. Further analyses included immunostaining for angiogenic and dermal maturation markers of interest. Collagen type I (Col-I; Abcam) and alpha-smooth muscle actin (α-SMA; Abcam) were used to evaluate the dermal remodelling, and transmission electron microscopy (TEM) was performed for ultrastructural validation.

### 2.12. Histological Analysis of Regenerated Skin

The fixed tissue samples were processed, embedded in paraffin, and sectioned into 5 µm-thick slices using a microtome (Leica Microsystems, Wetzlar, Germany). The sections were then subjected to a series of histological and immunohistochemical staining techniques, including haematoxylin and eosin (H&E; IHC World, Woodstock, MD, USA) and Masson’s trichrome (MT; Polysciences, Warrington, PA, USA), which were used to visualise the skin architecture, the collagen production, and the presence of collagen. Haematoxylin and eosin (H&E) staining was performed to evaluate the overall skin architecture and cellular details, allowing the identification of both nuclei and cytoplasm. MT staining was used to visualise the extracellular matrix (ECM) components, such as collagen, mucus (stained blue), keratin, muscle fibres, and the cell cytoplasm (stained red).

Briefly, the skin samples were cut into thin sections and embedded in paraffin.

The sections were treated with xylene solution for dewaxing, followed by rehydration using a graded ethanol series (100%, 95%, and 70%). For the H&E staining, the samples were stained with haematoxylin to visualise the nuclei, followed by eosin staining to counterstain the cytoplasm and extracellular components.

For the MT staining, the tissue was first deparaffinised and then placed in preheated Bouin’s fluid (Polysciences, USA) at 56 °C for 60 min and then cooled at room temperature for 10 min, as recommended by the manufacturer’s protocol. The slides were washed with running tap water, stained with Weigert’s iron haematoxylin for 5 min, and then rinsed thoroughly. The sections were subsequently stained with Biebrich scarlet-acid fuchsin solution, differentiated in phosphomolybdic/phosphotungstic acid solution for 10–15 min, and then counterstained with aniline blue for 5 min. After a final differentiation in acetic acid (1%) for 5 min, the sections were rinsed, dehydrated through increasing alcohol concentrations, cleared in xylene, and mounted with DPX.

The thickness of the epidermis layers of the regenerated wound was measured with the assistance of a histological assessment conducted by a blinded expert veterinarian from Universiti Putra, Malaysia, at 10 different random locations for each micrograph. This histological analysis enabled the evaluation of epidermal and dermal regeneration, collagen deposition, and ECM remodelling in the treated and control skin tissues.

### 2.13. Immunohistochemistry

Immunohistochemistry (IHC) staining was performed to assess the expression of collagen type I (Col-I; Abcam Cell Signalling Technology, UK) and alpha-smooth muscle actin (α-SMA; Abcam Cell Signalling Technology, UK) to evaluate the dermal layer maturation and identify the collagen type I production and myofibroblasts, respectively. To evaluate the extent of angiogenesis, the expression of CD31 (Abcam, Cambridge, UK) was studied. The tissue sections (5 μm) were prepared using a microtome. These sections were dewaxed with a series of xylene and alcohol solutions and treated with an antigen retrieval citrate buffer solution (pH 6.0; Sigma-Aldrich, USA) for 20 min at 100 °C. The tissue sections were blocked with 10% goat serum and incubated at 37 °C for 1 h to block the nonspecific binding sites. The tissue sections were then incubated overnight with primary antibodies at 4 °C. After washing with PBS, the tissue sections were incubated with secondary antibodies with different fluorophores at 37 °C for 1 h in the dark and counterstained with DAPI (Invitrogen, Carlsbad, CA, USA) at room temperature for 20 min. The images were captured using CLSM (Nikon, Japan). At 100× magnification, the basal dermal layer in the regenerated skin dermis was quantified by measuring the intensity (a.u.) at five different locations per sample.

### 2.14. Transmission Electron Microscopy (TEM)

The ultrastructure of regenerated skin, including tight junctions and collagen fibre organisation, was evaluated using TEM to assess skin maturity and structural integrity. Harvested skin samples (~1 mm length) were fixed in 4% glutaraldehyde (Bio-Rad, Hercules, CA, USA) at 4 °C overnight. Following three washes with 0.1 M sodium cacodylate buffer (Sigma-Aldrich), the samples were post-fixed with 4% osmium tetroxide (Sigma-Aldrich) for 1 h. After the additional washes with sodium cacodylate, the samples underwent a graded dehydration using acetone solutions ranging from 35% to 100%. The dehydrated tissues were infiltrated sequentially with acetone–resin mixtures (1:1 for 1 h and 1:2 for 2 h) and finally with 100% resin overnight. The polymerisation was carried out at 60 °C for 24–48 h. The tissue blocks were sectioned using an ultramicrotome (Leica, Teaneck, NJ, USA) to obtain semi-thin sections (0.5–1.0 μm) for toluidine blue staining and ultrathin sections (~90 nm) for TEM imaging. The ultrathin sections were stained with lead citrate and uranyl acetate and imaged using a FEI Tecnai G2 Spirit Biotwin TEM system (FEI, Waltham, MA, USA) at an accelerating voltage of 120 kV in bright field mode, with a magnification range of 8000× to 60,000×. The high-resolution images were acquired in TIFF format for detailed analysis.

### 2.15. Statistical Analysis

All the statistical and graphical analyses were performed using GraphPad Prism (version 9; GraphPad Software, Inc., San Diego, CA, USA). The quantitative data are presented as mean ± standard deviation (SD). In this study, nine adult male BALB/c mice were used, with each animal receiving two full-thickness excisional wounds, resulting in a total of 18 wounds. The wounds were randomly allocated into three experimental groups (C, B, and BE), yielding six wounds per group (*n* = 6). The individual wound was defined as the experimental unit for statistical analysis, as the wounds were created at anatomically separated dorsal sites and treated independently. This clarification has been added to avoid ambiguity in interpreting the statistical results. For comparisons among three or more groups involving a single independent variable, one-way analysis of variance (ANOVA) was applied, followed by Tukey’s multiple comparisons post hoc test where appropriate. When two independent variables were analysed, a two-way ANOVA was used to evaluate the potential interaction effects. For comparisons between the two groups, an unpaired Student’s *t*-test was performed. The statistical significance was defined as *p* < 0.05 (*), *p* < 0.01 (**), *p* < 0.001 (***), and *p* < 0.0001 (****). The image-based quantification was performed using ImageJ software (version 1.53t; NIH, USA). To ensure an unbiased interpretation, histological assessments were conducted by a blinded veterinary expert at Universiti Putra Malaysia.

## 3. Results

### 3.1. In Vivo Safety and Gross Wound Evaluation

Throughout the study, in vivo evaluation confirmed the safety and biocompatibility of all the treatments. The animals maintained stable health with no noticeable changes in their behaviour, vital signs, or body weight. No signs of pain, allergic reactions, excessive inflammation, infection, delayed healing, or abnormal scarring were observed at the wound sites. Regular monitoring ensured the consistent well-being of the animal and indicated a good treatment tolerance across all groups.

**Figure 2:**
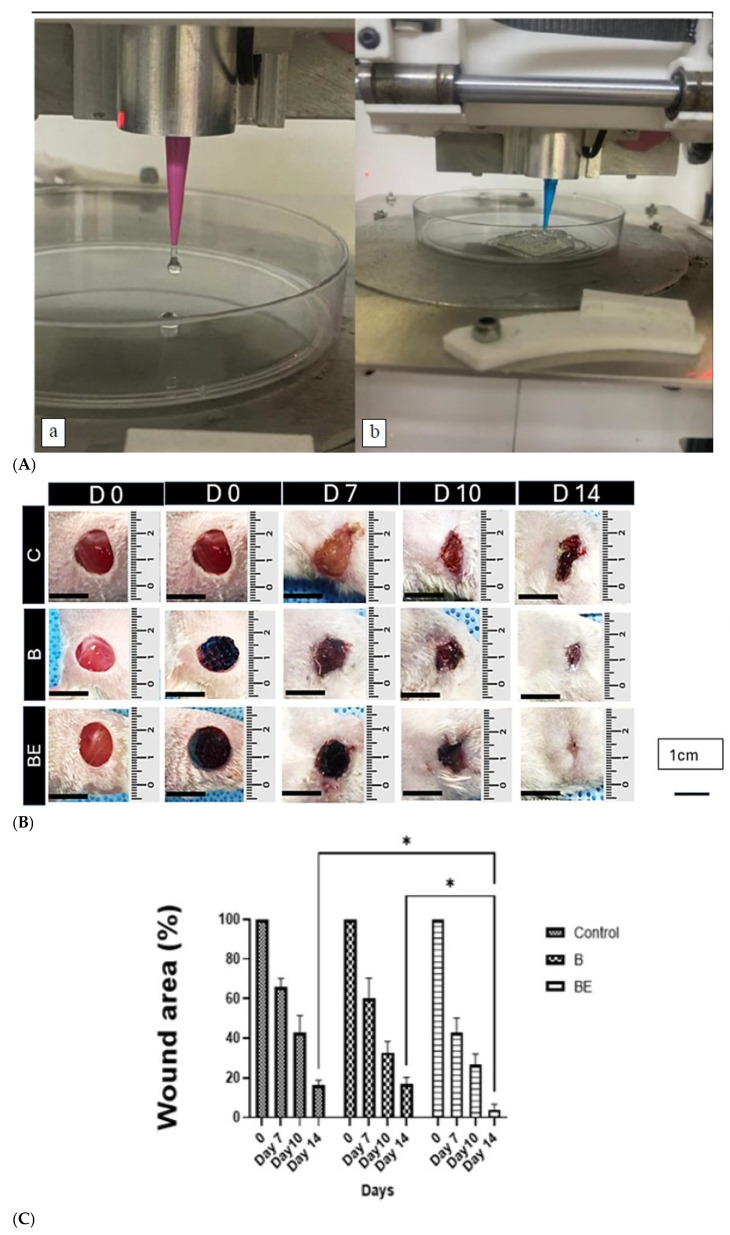
The evaluation of the wound healing progression in BALB/c mice treated with B and BE scaffolds. The normal saline served as the negative control (C group). (**A**), The extrusion-based 3D bioprinting of gelatin–genipin hydrogel scaffolds. (**a**) The initial extrusion from the nozzle to assess flow consistency at 22–24 °C. (**b**) The layer-by-layer printing to fabricate a grid-like scaffold. The scaffold design was created in Autodesk Fusion AutoCAD (v9.20.0.0), exported as STL, and processed in Simplify3D (v4.1) to define the nozzle path. (**B**), The representative gross images of the wounds from the treated (B and BE) and control (C) groups over the 14-day healing period. Both of the treatment groups exhibited complete re-epithelialisation by day 14, whereas the C group showed incomplete closure. Scale bar: 1 cm (black arrow). (**C**), The quantitative analysis of the wound area reduction (%) for all groups. The data are expressed as mean ± SD (*n* = 6 wounds per group). The statistical comparisons were performed using one-way ANOVA followed by Tukey’s post hoc test for comparisons between groups at each time point. The asterisks (*) indicate significant differences between the groups at the same time point (* *p* < 0.05). Abbreviations: C = control treated with normal saline, B = 10% gelatin crosslinked with 0.3% genipin (10% GECL), and BE = 10% GECL incorporated with 75 μg/mL small extracellular vesicles (sEVs).

Figure 2A shows the gross morphological evaluation of the wound healing from days 0 to 14, revealing progressive contraction in all groups, with no signs of infection or inflammation. By day 14, complete wound closure was observed in the group treated with the sEV-loaded hydrogel (BE), followed by the B group (hydrogel alone), whereas the control group (C) presented visible residual wound areas and scabbing, indicating delayed healing. Photographic monitoring at multiple time points confirmed these findings, with the BE group exhibiting the most rapid and complete skin regeneration. The quantitative analysis of the wound closure revealed significantly lower residual wound areas in the BE group than in the B and C groups on day 14 (3.89 ± 6.43% for BE vs. 14.75 ± 4.92% for B and 16.48 ± 5.40% for C), as shown in Figure 2B, supporting its superior regenerative efficacy. Although the wound sizes were comparable at early time points, the BE-treated wounds achieved near-complete closure by day 14, characterised by smooth, dry skin and the absence of crusts or scarring.

### 3.2. Histological Evaluation of Skin Regeneration

The histological analysis of the granulation tissue formation, dermis regeneration, and collagen production during wound healing in mice was performed. The regenerated skin was evaluated via H&E and Masson’s trichrome staining. The normal skin has a distinct bilayer structure characterised by a thin, keratinised epidermis and a highly cellular, mature, collagen-rich dermis. In contrast, the newly regenerated skin presented a thicker epidermal layer, a looser dermal matrix, and the presence of granulation tissue, indicating ongoing tissue remodelling.

#### 3.2.1. Haematoxylin and Eosin (H&E) Staining

**Figure 3:**
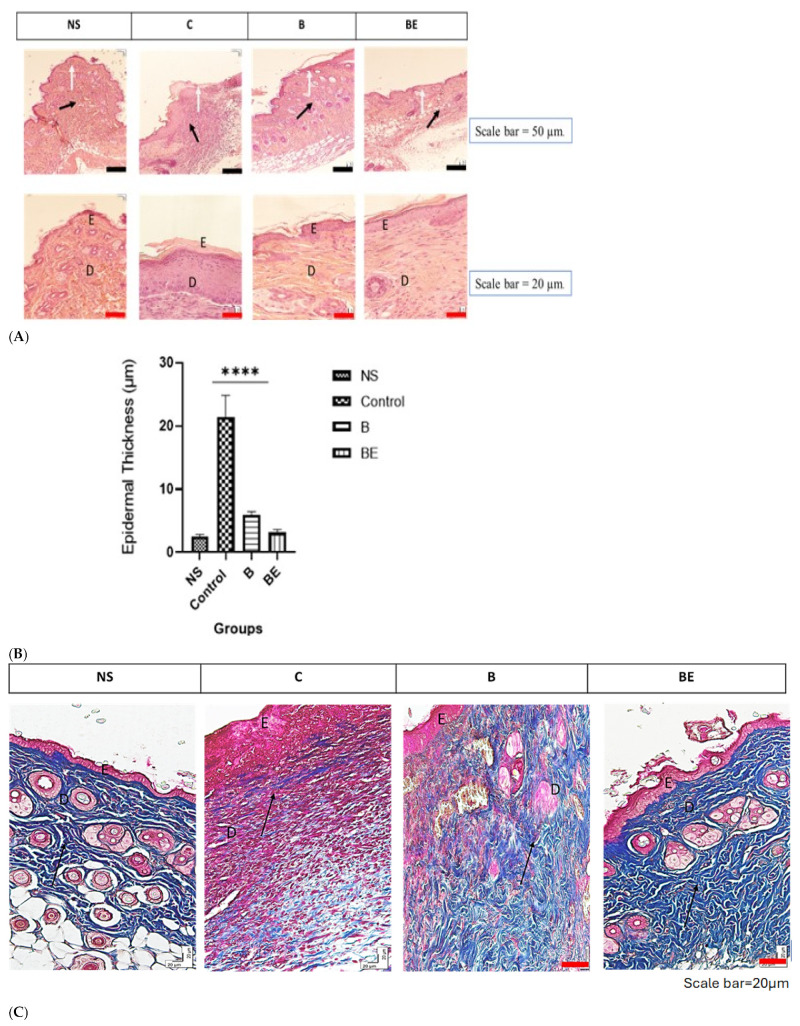
Histological evaluation of regenerated skin in BALB/c mice on day 14. (**A**) Haematoxylin and eosin staining showing epidermal thickening (hyperplasia) in regenerated tissue. The white arrow indicates the regenerated epidermis, and the black arrow indicates underlying granulation tissue and dermal remodelling. (**B**) Quantification of epidermal thickness across all groups (NS, B, BE, and C). The epidermal thickness in the C group was significantly higher than in the treated and NS groups (**** *p* < 0.0001). Data are presented as mean ± SD (μm) (*n* = 6). (**C**) Masson’s trichrome staining used to assess collagen deposition and organisation. Collagen fibres (blue) appeared more aligned in the treated groups (B and BE) compared with NS and C groups. Muscle fibres are shown in red/pink, collagen in blue, and cytoplasm in dark red/blue. Scale bars: black: 50 μm (20×) and red: 20 μm (40×). D = dermis; E = epidermis.

H&E staining was used to assess the skin regeneration by visualising the cellular and tissue architecture. The haematoxylin-stained nuclei were blue–purple, whereas the eosin-stained nuclei highlighted the cytoplasm and extracellular matrix in various shades of pink. By day 14, all the groups showed signs of re-epithelialisation with keratin layer formation, indicating ongoing healing. However, the control group (C) presented thick crusts and a disorganised epidermal architecture (Figure 3A). The quantitative analysis revealed significantly thinner epidermal layers in the B (5.93 ± 0.49 μm) and BE (3.16 ± 0.47 μm) groups than in the control (21.4 ± 3.49 μm) group, with the BE group closely approximating native skin (2.51 ± 0.32 μm), as demonstrated (Figure 3B).

#### 3.2.2. Masson’s Trichrome Staining

Masson’s trichrome staining was performed to assess the collagen deposition and organisation. In this method, the collagen fibres stain blue, the muscle appears red or pink, and the nuclei appear black or blue. On day 14, both the B and BE groups presented abundant collagen deposition in the papillary and reticular dermis. Although the staining does not distinguish the collagen types, the organised pattern and location are consistent with the typical collagen type I distribution. Compared with those in the control group, the collagen fibres in both treatment groups were more aligned and densely packed, which may indicate improved matrix organisation (Figure 3C).

### 3.3. In Vivo Biodegradation and Histological Evaluation of B and BE

To assess the in vivo biodegradation and biocompatibility of the developed hydrogels, histopathological studies were conducted on the surrounding skin tissues following the subcutaneous implantation. The shape and volume of the B and BE scaffolds were monitored over time to evaluate their degradation profiles. A gradual reduction in scaffold volume was observed, which was attributed to enzymatic activity in the bodies of the mice (e.g., lysozymes, collagenases, and lipases).

**Figure 4:**
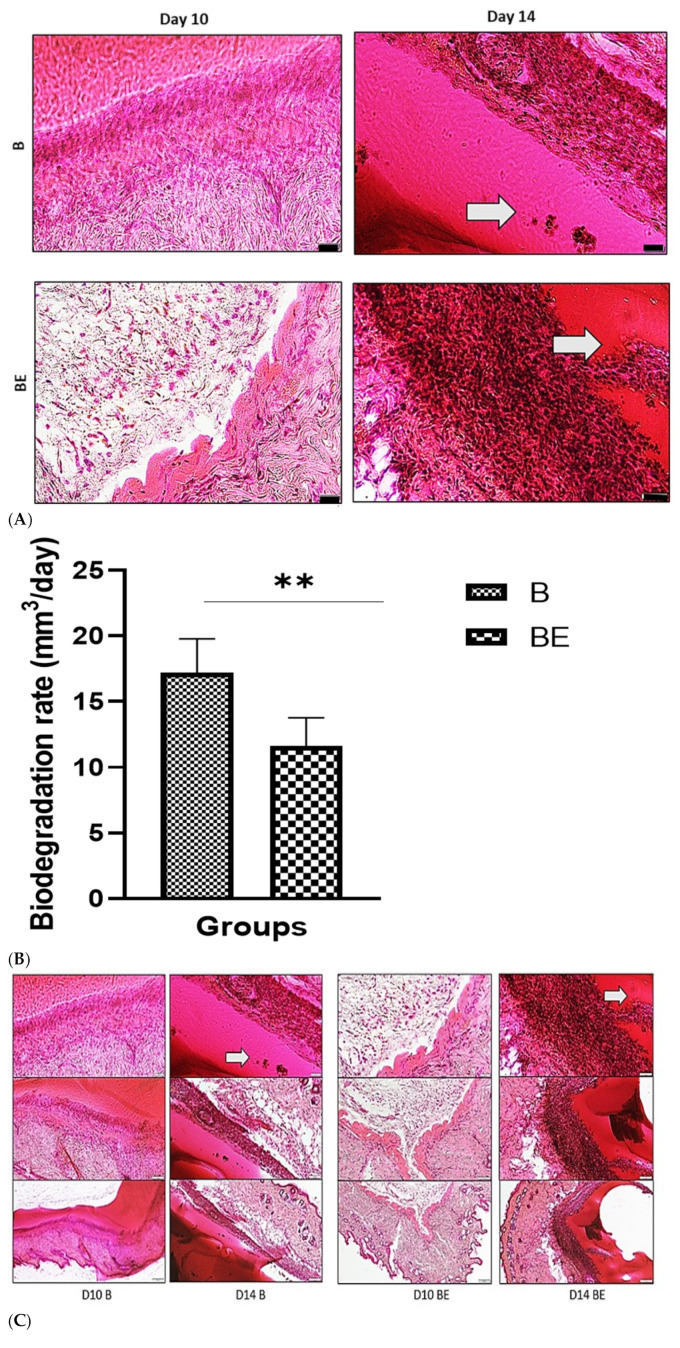
The histological analysis of in vivo biodegradation of the B and BE scaffolds over 14 days, showing in vivo biodegradation and cell infiltration (white arrows). The scaffolds appear as fibrous or matrix-like structures, whereas the infiltrating cells are visible as basophilic (purple/blue) nuclei within the scaffold pores. (**A**) shows the haematoxylin and eosin (H&E) staining of histological sections of the subcutaneously implanted scaffolds, which revealed the progressive biodegradation of the biomatrices. (**B**) shows the quantitative data of the biodegradation rates (mm^3^/day), which were calculated based on the volume measurements taken on days 0, 5, 7, 11, and 14 for both the B and BE scaffolds. (**C**) shows the degree of cellular infiltration into the scaffolds, as observed by the H&E staining, highlighting the differences between the hydrogels with (BE) sEVs and those without (B) sEVs at various time points. The scale bar is 50 μm. B = 10% gelatin crosslinked with 0.3% genipin (10% GECL) and BE = 10% GECL incorporated with 75 μg/mL small extracellular vesicles (sEVs). The data are expressed as the means ± standard deviations (*n* = 3). The statistical significance is denoted as ** *p* < 0.01.

Figure 4A shows the haematoxylin and eosin (H&E) staining of tissue sections collected on day 14. Notably, fibroblast accumulation occurs at the scaffold implantation site. These findings suggest that the scaffolds provided a favourable matrix for fibroblast migration, proliferation, and granulation tissue formation, which are key steps in wound healing.

A quantitative assessment of degradation (Figure 4B) was performed by measuring the scaffold dimensions on days 0, 5, 7, 11, and 14. The B scaffold degraded at a rate of 17.19 ± 2.601 mm^3^/day, whereas the BE scaffold showed a slightly lower degradation rate of 11.63 ± 2.138 mm^3^/day. Although the BE scaffolds appeared to degrade more slowly, the difference was not statistically significant.

The histological analysis further confirmed the biocompatibility, as shown by the H&E-stained sections. At 14 days post-implantation, fibroblasts were observed at the hydrogel implantation site, demonstrating the scaffold’s ability to support cell growth, proliferation, and migration. Additionally, the hydrogel attracted inflammatory cells and fibroblasts, successfully facilitating granulation tissue development (Figure 4C).

### 3.4. Immunohistochemical Staining of Newly Formed Skin

**Figure 5:**
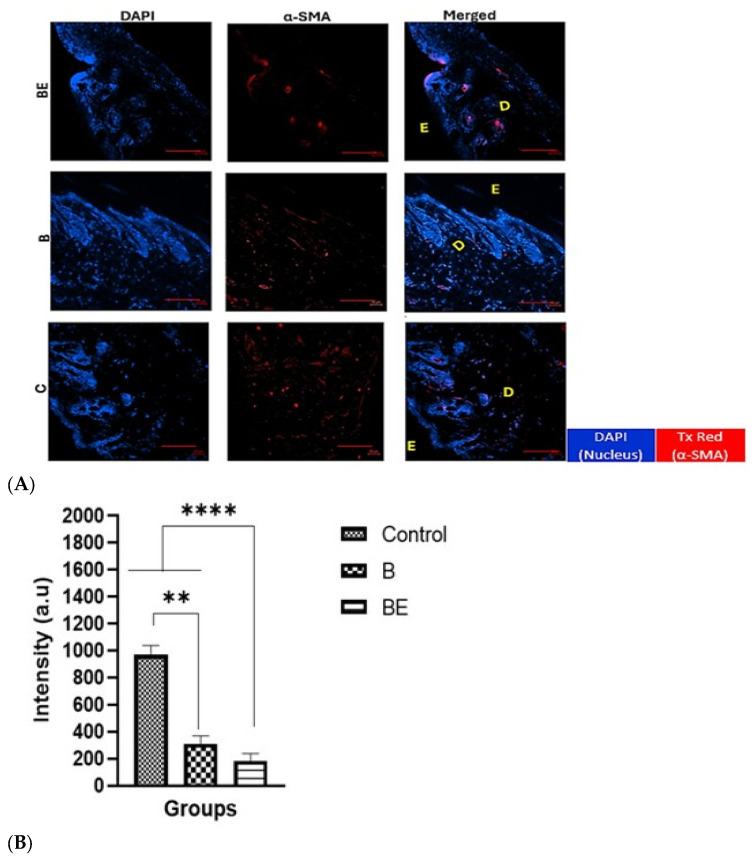

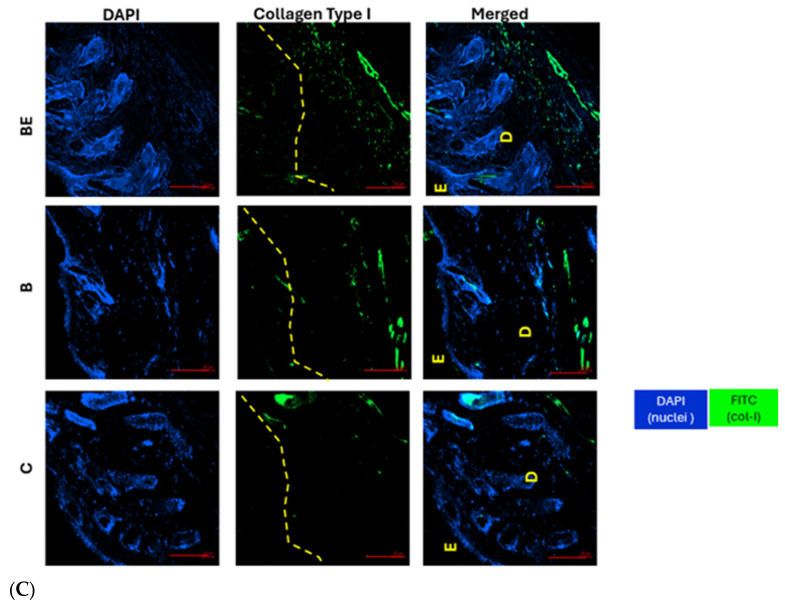
The immunohistochemical (IHC) staining of the regenerated dermal layer on day 14 posttreatment. (**A**) shows the expression of α-smooth muscle actin (α-SMA, red), a marker of myofibroblasts, which were counterstained with DAPI (blue) to visualise the nuclei. The letter E represents epidermal, and the letter D represents dermal. The yellow line indicates the boundary between the epidermis and the dermis. The scale bar is 50 μm. (**B**) shows the results of the quantitative analysis of α-SMA intensity (arbitrary units, a.u.) in the B, BE, and control (C) groups. (**C**) shows the collagen type I (Col-I) expression (green), which is indicative of dermal matrix deposition activity in the dermis and was also counterstained with DAPI (blue). Col-I expression was observed in all the experimental groups. The letter E represents epidermal, and the letter D represents dermal. The scale bar is 50 μm. *(n* = 6). The data are presented as the means ± standard deviations (*n* = 6). The statistical significance: ** *p* < 0.01, and **** *p* < 0.0001. Abbreviations: B = 10% gelatin crosslinked with 0.3% genipin (GECL); BE = GECL with 5 μg/mL UC-MSC-derived sEVs; and C = untreated control group.

The IHC analysis was conducted to assess extracellular matrix deposition and myofibroblast activity in the regenerated dermal tissue. Collagen type I (Col-I) and alpha-smooth muscle actin (α-SMA) staining revealed distinct expression patterns across the groups. The expression of α-SMA, a marker of myofibroblast activity associated with wound contraction, was most prominent in the C group, with reduced levels in the B group and notably lower expression in the BE-treated skin (Figure 5A). The quantitative analysis of α-SMA staining intensity confirmed this pattern, with the BE group exhibiting the lowest signal (184 ± 56.0 a.u.), which was significantly less intense than that of the B (313 ± 56.7 a.u.) and C (970 ± 69.6 a.u.) groups, as shown in Figure 5B. As shown in Figure 5C, Col-I was positively expressed in all the samples, indicating active ECM production, with stronger and more organised deposition observed in the B and BE groups than in the control group (C), as demonstrated by Masson’s trichrome staining (Figure 3C). These findings support enhanced dermal remodelling in the treated wounds.

Interestingly, in the BE group (Figure 5A), α-SMA was also observed surrounding the blood vessels, forming a ring-like pattern indicative of smooth muscle cell involvement in vascular maturation rather than widespread myofibroblast activation. This distinction implies that sEV-functionalised hydrogels not only support tissue regeneration but also regulate fibroblast behaviour, minimising undesirable contraction while promoting angiogenesis.

**Figure 6:**
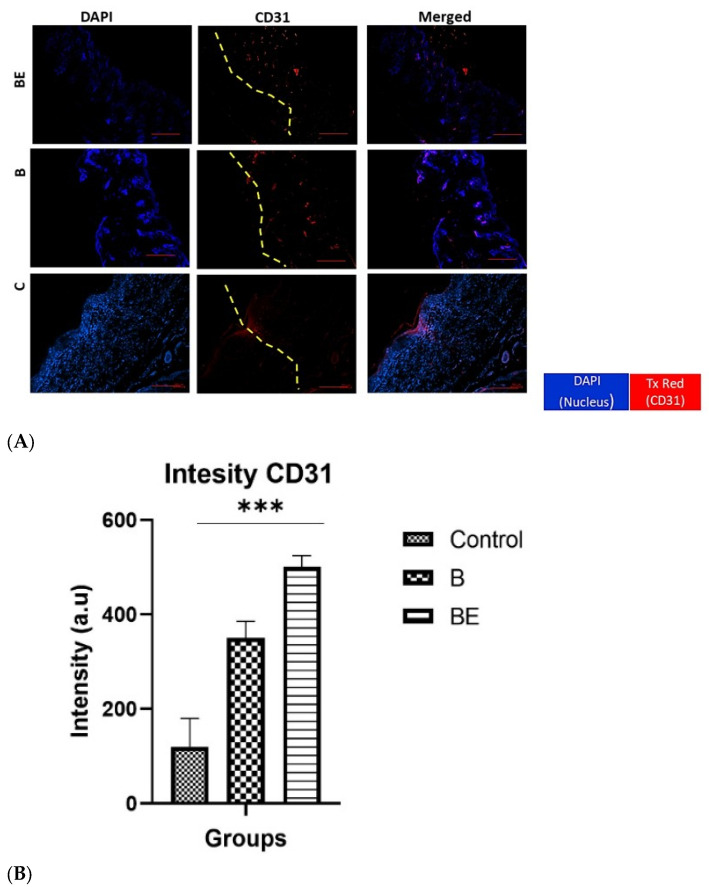
The immunohistochemical (IHC) staining of the regenerated dermal layer on day 14 posttreatment. (**A**) shows the expression of CD31 (red), a marker of angiogenesis, which was counterstained with DAPI (blue) to visualise the nuclei. The yellow line indicates the boundary between the epidermis and dermis. The scale bar is 50 μm. (**B**) shows the results of the quantitative analysis of the CD31 intensity (arbitrary units, a.u.) in the B, BE, and control (C) groups. The data are presented as the means ± standard deviations (*n* = 6). The statistical significance: *** *p* < 0.001. Abbreviations: B = 10% gelatin crosslinked with 0.3% genipin (GECL); BE = GECL with 75 μg/mL UC-MSC-derived sEVs; and C = untreated control group.

To evaluate angiogenesis at the wound site, immunohistochemical (IHC) staining was performed on day 14 post-wounding using anti-CD31, which is a well-established marker of endothelial cells, along with DAPI for nuclear counterstaining. As shown in Figure 6A, clear differences were observed in CD31 expression among the experimental groups.

The untreated control group (C) displayed weak CD31 staining, which is indicative of minimal neovascularisation. In contrast, the biomaterial-only group (B) presented a moderate increase in CD31-positive staining. The quantitative analysis of CD31-positive areas, which was based on three high-power fields per sample, corroborated these findings. The BE group presented the highest vascular density (500.8 ± 23.99 a.u.), which was significantly greater than that of both the B group (350.3 ± 35.65 a.u.) and the control group (120.3 ± 59.55 a.u.). These results provide strong evidence that the BE formulation significantly enhances neovascularisation during wound healing (Figure 6B).

### 3.5. Ultrastructure of Newly Regenerated Skin

**Figure 7:**
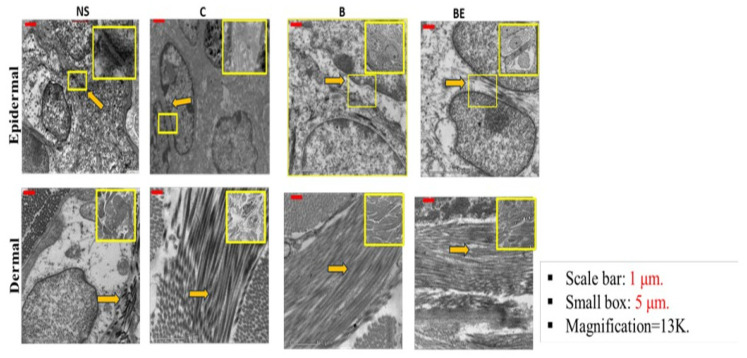
The transmission electron micrographs of the epidermal and dermal structures in the normal skin, control (saline-treated), B, and BE groups. The images show epidermal tight junctions (red arrows) and dermal collagen fibres (yellow arrows) across the treated and nontreated (c) groups. The characteristic striations are visible. The tight junction components associated with keratin filaments are highlighted in the small yellow inset box. The scale bars represent 1 μm, and the yellow inset boxes indicate regions of 5 μm. The images were acquired at a magnification of 13,000×. The images were captured at 13,000× magnification.

The transmission electron microscopy (TEM) revealed a more compact epidermal architecture and well-defined tight junctions in both the B and BE groups than in the poorly organised epidermis and an absence of tight junctions in the control group (C) (Figure 7). The basal membranes were also observed in the treated groups, indicating better epidermal-dermal attachment. In the dermis, collagen fibrils with characteristic cross-patterns, indicative of type I collagen, were present in all the groups but were less dense in the control group, suggesting a delayed remodelling. The dermal–epidermal junction (DEJ) could not be identified in any group. Collectively, these findings suggest an enhanced ultrastructural regeneration in the treated wounds.

## 4. Discussion

Full-thickness wound repair is often limited by delayed re-epithelialisation, disorganised collagen deposition, and insufficient angiogenesis. Cell-free therapies using mesenchymal stem cell-derived small extracellular vesicles (MSC-sEVs) have emrged as a promising strategy, delivering regenerative bioactivity without the risks associated with cell transplantation [14]. Incorporating sEVs into hydrogels provides a supportive matrix for a localised, sustained release, which has been confirmed in vitro using the same GECL formulations [12]. While a free-sEV group was not included in the present study, prior work has demonstrated that the scaffold provides a gradual and prolonged release of sEVs, ensuring an enhanced local bioavailability. In this study, 10% genipin-crosslinked gelatin (GECL) hydrogels were loaded with hUCMSC-derived sEVs that retained their morphology and marker expression in accordance with MISEV 2023 guidelines.

In a murine full-thickness wound model, sEV-loaded hydrogels (BE group) significantly accelerated the wound closure, re-epithelialisation, and dermal remodelling compared to the hydrogel-only and control groups. The enhanced healing reflects the synergistic combination of sustained sEV delivery and scaffold support, coordinating multiple regenerative processes. Importantly, this work demonstrates for the first time the in vivo therapeutic efficacy of GECL scaffolds with hUCMSC-sEVs in full-thickness wounds, highlighting their potential to improve angiogenesis, fibroblast activation, and extracellular matrix remodelling. The consideration of the EV dose and scaffold properties further underscores the translational relevance of this platform while acknowledging the limitations relative to the alternative hydrogel systems and crosslinking approaches [14,15].

One of the best outcomes was the markedly enhanced extracellular matrix (ECM) remodelling that was observed in the BE group. The histological and Masson’s trichrome staining revealed denser, better-aligned collagen fibres than those in the controls did, which is consistent with the previous studies showing that sEVs stimulate fibroblast proliferation, promote collagen synthesis, and limit excessive matrix degradation [16,17,18,19]. Balanced ECM remodelling is critical for functional regeneration, since an excessive or disorganised deposition predisposes the tissue to fibrotic scarring, whereas well-structured fibres contribute to tissue strength and elasticity.

In parallel, angiogenesis was strongly promoted in the BE group, as evidenced by the increased CD31 expression and the formation of the well-organised microvessels. The sustained vascularisation is indispensable for supplying oxygen and nutrients to regenerating tissue, and sEVs are known to transfer proangiogenic miRNAs and growth factors such as VEGF [6,16,20]. These findings align with the previous reports and further suggest that the GECL hydrogel acted as a protective reservoir, prolonging the sEV bioactivity at the wound site.

Interestingly, the α-SMA expression in the BE group was transiently elevated, reflecting a controlled myofibroblast activation. This is advantageous in early wound healing, where myofibroblasts drive contraction but become harmful if prolonged, as it can lead to fibrosis. In our study, the α-SMA expression decreased during the later stages, suggesting that sEVs modulate the fibroblast-to-myofibroblast transition and reduce the risk of hypertrophic scarring [15,17].

The ultrastructural analysis further confirmed the epidermal barrier restoration. The TEM revealed the compact keratinocyte junctions, desmosomes, and intact dermal epidermal attachment in the BE group, highlighting not only an accelerated re-epithelialisation but also an improved barrier integrity, an often overlooked yet essential determinant of wound resolution [21,22]. A limitation of this study is the absence of quantitative ultrastructural analysis, as the TEM evaluation was primarily qualitative. Incorporating quantitative metrics could provide deeper mechanistic insights and will be considered in future studies.

Another important aspect was the degradation profile of the GECL scaffold, which closely matched the progression of the wound healing. The gradual resorption enabled tissue ingrowth while maintaining a temporary structural support. Hydrogels are widely recognised as promising drug delivery systems due to their ability to regulate the release of encapsulated therapeutics through their network structure, crosslinking density, and biodegradability. The previous studies have demonstrated that extracellular vesicle (EV)-loaded hydrogels can enable a sustained and controlled release during hydrogel degradation, thereby enhancing the EV stability and therapeutic efficacy [23]. In this study, the sustained release behaviour of the sEVs from the GECL scaffold is therefore inferred based on these established properties rather than directly quantified. This behaviour distinguishes the genipin-crosslinked gelatin from the faster-degrading natural hydrogels, which often lack sufficient in vivo stability [24,25]. A limitation of this study is that the hydrogel biodegradation was expressed as an average daily rate, which may not fully reflect its nonlinear kinetics. Future work will incorporate a time-course analysis and kinetic modelling to provide a more comprehensive understanding of the degradation behaviour.

By day 14, the wounds that were treated with BEs achieved nearly complete closure without crust formation, demonstrating an effective re-epithelialisation and a seamless integration with surrounding tissue. In contrast, the control wounds displayed persistent scabbing and incomplete closure, whereas the hydrogel-only wounds exhibited delayed healing. These results underscore the indispensable role of sEVs in attractive scaffold-mediated regeneration. The sEV-only group was not included because of the rapid clearance and poor retention of the free sEVs in vivo. Instead, our approach focused on the sustained, localised sEV delivery through hydrogel incorporation, which more closely mimics a clinical application and significantly improves the therapeutic efficacy.

Compared with the other exosome–hydrogel models, the BE group achieved faster wound closure by day 14, with closure occurring around day 18 [18]. This superior performance is likely attributable to the synergistic combination of the sEVs with the GECL scaffold, which enhances the delivery efficiency, prolongs the release kinetics, and facilitates the cellular responses such as fibroblast migration and angiogenesis. These results align with the earlier studies demonstrating that hydrogels, such as GelMA [26] or Alg-EXO composites [17], prolong sEV activity and accelerate wound repair. Together, our findings highlight the translational advantage of GECL-sEV hydrogels in terms of reaching more rapid and functionally robust healing outcomes. Importantly, the present findings extend beyond the prior in vitro observations by demonstrating a coordinated modulation of key wound healing processes in vivo, including angiogenesis, controlled myofibroblast activation, and organised collagen deposition, highlighting the functional bioactivity of the GECL–sEV system within a complex physiological environment.

Together, this study underscores the synergistic benefits of integrating a mechanically stable, biodegradable hydrogel with bioactive sEVs. Unlike a direct sEV injection, which suffers from rapid clearance, or cell transplantation, which involves regulatory and safety concerns, our strategy offers a cell-free, localised, and sustained delivery system that maximises the therapeutic efficacy while minimising the risks.

Nonetheless, certain limitations should be acknowledged. The current work focused on short-term healing in a murine model; long-term functional recovery, immune modulation, and evaluation in large animal models remain to be investigated. Additionally, the optimisation of the sEV dosing and the release of kinetics will be crucial for clinical translation. We also acknowledge that the molecular-level mechanistic pathways were not directly investigated; however, the observed tissue-level responses provide a strong foundation for future mechanistic studies.

## 5. Conclusions

A 3D bioprinted genipin-crosslinked gelatin (GECL) hydrogel functionalised with hUCMSC-sEVs was developed and shown to significantly enhance full-thickness wound healing. The synergistic effect of the hydrogel scaffold and sEVs promoted re-epithelialisation, organised collagen deposition, angiogenesis, and cell proliferation. These findings highlight the potential of sEV-functionalised hydrogels as effective cell-free therapeutic strategies for wound regeneration and their translational potential for future clinical applications.

## Data Availability

The original contributions presented in this study are included in the article. Further inquiries can be directed to the corresponding author.

## References

[1] Zandi N., Dolatyar B., Lotfi R., Shallageh Y., Shokrgozar M.A., Tamjid E., Annabi N., Simchi A. (2021). Biomimetic nanoengineered scaffold for enhanced full-thickness cutaneous wound healing. Acta Biomater..

[2] Zhong W., Meng H., Ma L., Wan X., Chen S., Ma K., Lu L., Su J., Guo K., Jiang Y. (2024). Hydrogels loaded with MSC-derived small extracellular vesicles: A novel cell-free tissue engineering system for diabetic wound management. View.

[3] Toh W.S., Lai R.C., Zhang B., Lim S.K. (2018). MSC exosome works through a protein-based mechanism of action. Biochem. Soc. Trans..

[4] Portela R., Leal C.R., Almeida P.L., Sobral R.G. (2019). Bacterial cellulose: A versatile biopolymer for wound dressing applications. Microb. Biotechnol..

[5] Gounden V., Singh M. (2024). Hydrogels and wound healing: Current and future prospects. Gels.

[6] Ji S., Zhao Y., Zhai X., Wang L., Luo H., Xu Z., Dong W., Wu B., Wei W. (2023). A dual-crosslinked hydrogel based on gelatin methacryloyl and sulfhydrylated chitosan for promoting wound healing. Int. J. Mol. Sci..

[7] Zawani M., Maarof M., Tabata Y., Motta A., Fauzi M.B. (2022). Quercetin embedded gelastin injectable hydrogel as provisional biotemplate for future cutaneous application: Optimization and in vitro evaluation. Gels.

[8] Smandri A., Nordin A., Hwei N.M., Chin K.Y., Abd Aziz I., Fauzi M.B. (2020). Natural 3D-printed bioinks for skin regeneration and wound healing: A systematic review. Polymers.

[9] Javaid M., Haleem A. (2021). 3D bioprinting applications for the printing of skin: A brief study. Sens. Int..

[10] Taghdi M.H., Muttiah B., Chan A.M.L., Fauzi M.B., Law J.X., Lokanathan Y. (2024). Exploring Synergistic Effects of Bioprinted Extracellular Vesicles for Skin Regeneration. Biomedicines.

[11] Jeong J.-W., Park D.-J., Kim S.-C., Kang H.W., Lee B., Kim H.-W., Kim Y.-M., Linh N.V., Jung W.-K. (2025). Wound healing effect of fucoidan-loaded gelatin/oxidised carboxymethyl cellulose hydrogel. Int. J. Biol. Macromol..

[12] Taghdi M.H., Al-Masawa M.E., Muttiah B., Fauzi M.B., Law J.X., Zainuddin A.A., Lokanathan Y. (2025). Three-dimensional bioprinted gelatin–genipin hydrogels enriched with hUCMSC-derived small extracellular vesicles for regenerative wound dressings. Polymers.

[13] Chea P.S., Low K.C., Mohidin N., Bariah M., Maung M., Azian A. (2007). Ketamine–xylazine/tiletamine–zolazepam prolonged anesthesia in cynomolgus monkeys. Online J. Vet. Res..

[14] Huang J., Xiong J., Yang L., Zhang J., Sun S., Liang Y. (2021). Cell-free exosome-laden scaffolds for tissue repair. Nanoscale.

[15] Zhang W., Huang X. (2023). Stem cell-based drug delivery strategy for skin regeneration and wound healing: Potential clinical applications. Inflamm. Regen..

[16] Liu J., Yan Z., Yang F., Huang Y., Yu Y., Zhou L., Sun Z., Cui D., Yan Y. (2021). Exosomes derived from human umbilical cord mesenchymal stem cells accelerate cutaneous wound healing by enhancing angiogenesis through delivering angiopoietin-2. Stem Cell Rev. Rep..

[17] Shafei S., Khanmohammadi M., Heidari R., Ghanbari H., Taghdiri Nooshabadi V., Farzamfar S., Akbariqomi M., Sanikhani N.S., Absalan M., Tavoosidana G. (2020). Exosome loaded alginate hydrogel promotes tissue regeneration in full-thickness skin wounds: An in vivo study. J. Biomed. Mater. Res. Part A.

[18] Zhang Y., Zhang P., Gao X., Chang L., Chen Z., Mei X. (2021). Preparation of exosomes encapsulated nanohydrogel for accelerating wound healing of diabetic rats by promoting angiogenesis. Mater. Sci. Eng. C Mater. Biol. Appl..

[19] Sabarees G., Vishvaja S., Raghuraman S., Velmurugan V., Alagarsamy V., Solomon V.R., Tamilarasi G.P. (2025). Collagen-based nanofibers: Revolut therapeutics for impaired wound healing. Int. J. Polym. Mater. Polym. Biomater..

[20] Zhao D., Yu Z., Li Y., Wang Y., Li Q., Han D. (2020). GelMA combined with sustained release of HUVECs derived exosomes for promoting cutaneous wound healing and facilitating skin regeneration. J. Mol. Histol..

[21] Arnold K.A., Moran M.C., Shi H., Van Vlijmen-Willems I.M.J.J., Rodijk-Olthuis D., Smits J.P.H., Brewer M.G. (2024). CLDN1 knock out keratinocytes as a model to investigate multiple skin disorders. Exp. Dermatol..

[22] Byun K.A., Kim H.M., Oh S., Batsukh S., Son K.H., Byun K. (2024). Radiofrequency treatment attenuates age-related changes in dermal–epidermal junctions of animal skin. Int. J. Mol. Sci..

[23] Li Z., Liu J., Song J., Yin Z., Zhou F., Shen H., Wang G., Su J. (2024). Multifunctional hydrogel-based engineered extracellular vesicles delivery for complicated wound healing. Theranostics.

[24] Fan L., Liu C., Chen X., Zheng L., Zou Y., Wen H., Guan P., Lu F., Luo Y., Tan G. (2022). Exosomes-loaded electroconductive hydrogel synergistically promotes tissue repair after spinal cord injury via immunoregulation and enhancement of myelinated axon growth. Adv. Sci..

[25] Peñuelas N., Laguna A., Vila M. (2024). Hematoxylin and Eosin (H&E) Staining v1. Protoc. Exch..

[26] Chen Y.Q., Zhou Y.Q., Wei Q., Xie X.Y., Liu X.Z., Li D.W., Shen Z.A. (2024). Effects of gelatin methacrylate anhydride hydrogel loaded with small extracellular vesicles derived from human umbilical cord mesenchymal stem cells in the treatment of full-thickness skin defect wounds in mice. Zhonghua Shao Shang Yu Chuang Mian Xiu Fu Za Zhi.

